# Experiences of mothers of NICU preterm infants in milk management out of the hospital: a qualitative study

**DOI:** 10.1186/s13006-022-00540-2

**Published:** 2022-12-31

**Authors:** Rui Yang, Danqi Chen, Hua Wang, Xinfen Xu

**Affiliations:** 1 School of Nursing, Capital Medical University, Beijing, China; 2 Women’s Hospital, School of Medicine, Zhejiang University, Zhejiang, Hangzhou China; 3 Haining Maternal and Child Health Hospital, Branch of Women’s Hospital, School of Medicine, Zhejiang University, Hangzhou, China

**Keywords:** NICU, Preterm infants, Human milk management

## Abstract

**Background:**

Human milk is important for the health and development of preterm infants. China’s neonatal intensive care units (NICUs) have adopted the management system of maternal–infant separation. Human milk received and used by NICUs is managed by the infants’ families in the out-of-hospital environment. There is scant publication on mothers’ opinions on out-of-hospital human milk management. This study aimed to explore the experiences of Chinese mothers providing their infants in the NICUs with human milk expressed outside of the hospital.

**Methods:**

Semi-structured interviews were conducted with 23 participants recruited from June 2020 to November 2020, who transported their human milk to the human milk bank of Women’s Hospital School of Medicine Zhejiang University during the hospitalization of their preterm infants. This study adopted a qualitative research approach with thematic analysis.

**Results:**

Three main themes were identified: 1) awareness of human milk management and a willingness to adopt it; 2) lack of standardization regarding expressing, storing, and transporting expressed human milk; and 3) the need for more external support. Theme 2 additionally has three sub-themes: I) differentiation of preparations before human milk expression; II) differentiation of devices for human milk expression; and III) insufficient knowledge and understanding.

**Conclusions:**

In this study, all participants who received health education showed enthusiasm for participating in out-of-hospital human milk management. However, most participants had questions during the implementation process. Medical staff should provide professional and continuous external support to support mothers in implementing human milk management.

**Supplementary Information:**

The online version contains supplementary material available at 10.1186/s13006-022-00540-2.

## Background

Human milk is the ideal food for infants [1, 2]. International health organizations, including the World Health Organization (WHO), the United Nations Children’s Fund (UNICEF), the American Academy of Pediatrics, and the Japan Pediatric Society actively advocate human milk feeding in neonatal intensive care units (NICUs) to reduce the incidence of complications of preterm infants [3–5]. Human milk has become the preferred choice for NICU-hospitalized infants due to its benefits [6]. The European Society for Paediatric Gastroenterology, Hepatology, and Nutrition also recommends that preterm formula should be used only when human milk is not available [7]. There is increasing evidence that human milk can reduce the incidence of complications in preterm infants—for example, necrotizing enterocolitis [8–10] and promote the establishment and development of the gut microbiome [11].

In the first 3 months of lactation, fresh human milk (within 4 hours of expression) contains higher caloric, lipid and lactoferrin content than frozen human milk [12]. Therefore, the use of fresh human milk is more beneficial to preterm infants [13]. In China’s NICUs, where maternal–infant separation is implemented, priority is given to fresh human milk for preterm infants who are receiving human milk. However, the first month after giving birth is known as the *sitting month* in China. Women are highly recommended to stay in bed for the majority of this period and to not be away from home. Because of this tradition and the mother-infant separation management system in the NICU, fresh human milk can only be obtained by transporting human milk to the NICU. Human milk received and used by the NICU is managed by the infants’ families in the out-of-hospital environment. This includes milk expression, transportation, storage, and processing.

Environmental factors and the manner in which human milk is handled can affect human milk’s composition [14]. During the storage and processing of expressed human milk, there are risks of bacterial contamination, decreased immunological activity, and reduced nutritional potential [15]. The Sri Lankan Health Agency strongly recommends against the use of pumps for expressing breast milk, a hygiene concern is that pumps are considered a potential source of harmful bacteria [16]. In a survey in Melbourne, Australia, it was found that only 54% of mothers correctly understood the information on the refrigeration times of expressed human milk [17]. The study also found that the surface temperature of refrigerated fresh human milk transported to the NICU was higher than the temperature (1 °C–4 °C) recommended by the hospital. Some mothers did not put refrigerated human milk in insulated food containers, resulting in the temperature of these samples of human milk being higher than it would have been in the insulated food container [17]. Changes in temperature may reduce biologically active factors as well as the nutritional and microbiological value of fresh human milk [18, 19]. Inappropriate practices during the human milk management process may bring hidden dangers to human milk safety.

In 2018, Chinese researchers found that most (66%) hospitals had no lactation rooms and many (81%) NICUs provided neither hospital-grade milk pumps nor disposable milk storage bags [20]. Mothers could only express milk at home and then transport human milk to the hospital via milk storage bags or bottles for the infants. This poses challenges to the human milk feeding and management of hospitalized preterm infants [21, 22]. Paying attention to out-of-hospital human milk management is not only important for ensuring the quality of human milk during the hospitalization of preterm infants, but it is also important for mothers who have to express human milk as well as their families. Although a health education manual on human milk management is provided to mothers, which covers hand washing, human milk expression (including how to pump human milk, pumping frequency, breast pump cleaning, etc.), human milk storage and transportation, as well as breastfeeding knowledge. However, as the primary implementer of milk management, mother’s experiences remain to be studied.

Identifying the experiences of mothers who are conducting human milk management outside the hospital is important for providing support for the continuation of human milk expression. This study aimed to explore the experiences of Chinese mothers providing their newborns in the NICU with human milk expressed outside the hospital.

## Methods

### Design

This was a descriptive qualitative study. This research design enabled us to explore the experiences of Chinese mothers providing their newborns in the NICU with human milk expressed outside of the hospital.

### Setting

The study was conducted in the level 3 NICU preterm infant ward of Women’s Hospital School of Medicine Zhejiang University in China that has its own human milk bank, 105 beds, and a staff of more than 100 pediatricians and nurses.

In 2017, China issued expert recommendations for the operation and management of human milk banks in mainland China that included guidance for mothers’ education [23]. Lactation support providers and NICU nurses provide human milk management education when preterm infants are admitted to the hospital.

### Participants

The inclusion criteria of participants were mothers whose infants were hospitalized in the NICU and were willing to express and transport human milk to the NICU. The exclusion criteria were mothers with contraindications to breastfeeding and those with mental disorders, speech disorders, and visual or hearing impairment.

In order to attempt to maximize differences and obtain the required information, purposive sampling with a maximum variation sampling strategy was used, so we tried to include participants with different situations, including but not limited to maternal age, delivery method, gestational age, infant birth weight. Twenty-eight mothers received information about the study and five declined to participate. A total of 23 interviews were conducted, and the recruitment of participants stopped when no new information was obtained.

### Data collection

Participants were recruited from June 2020 to November 2020. Interviews were conducted at an agreed time with mothers, usually after the mother transported human milk. The research team comprised two doctoral researchers, NICU head nurse and a senior researcher. After obtaining the participants’ written consent, the doctoral researchers collected baseline demographic information from the participants and conducted semi-structured, private interviews, using the interview guide and making audio recordings of the interviews. Before commencing the study, the team members reviewed the principles of qualitative study and received systematic training in the theories of patient safety culture, aiming to establish a trusting relationship with participants and ensuring the smooth collection of data. Each participant was interviewed once. The interview guide was originally formulated by the two doctoral researchers and reviewed and revised by the NICU head nurse, and mock interviews were conducted to limit question bias. The interviews varied from 35 minutes to 50 minutes and were conducted in a small conference room provided by the NICU human milk bank.

### Data analysis

The interviewers transcribed the recordings verbatim within 48 hours after the interview. Memo and reflection diaries were written when necessary to adjust the next interview strategy and improve the authenticity and accuracy of the data. Thematic analysis was applied to the data. A systematic process was used to search across the dataset to identify, analyze, and report repeated patterns [24]. The data were analyzed by two researchers, who followed a six-stage analysis [25]: 1) familiarizing themselves with the data; 2) generating initial codes; 3) searching for themes; 4) reviewing themes; 5) defining and naming themes; and 6) producing the report.

The interviewers read and familiarized themselves with the transcribed text, and codes were generated for the latent and manifest content. Themes were identified among the codes, and when there were different opinions, discussions were held until agreement was reached. After transcribing and coding 20 recordings, they agreed that theme saturation might have been reached. They conducted 3 more interviews and found that no new codes or themes appeared, thus verifying theme saturation.

To assess trustworthiness of the study, the criteria proposed by Lincoln and Guba [26] was used. These criteria including credibility, confirmability, dependability and transferability. To ensuring the credibility in this current study, there was constant engagement with the data and the extracted codes and themes were shared with some of the participants and their opinions were sought (member-checking). To determine data confirmability, the NICU head nurse checked the study process and findings. To determine the dependability of the findings, all of the activities including the study process and achievement of the findings were recorded carefully, and an audit trail on the research process was provided. To determine data transferability, the findings were shared with the senior researcher that did not participate in the study and she was invited to give feedback of the transferability of the study. Two bilingual researchers verified the accuracy of the translated transcripts.

### Ethics

The study was approved by the Women’s Hospital School of Medicine Zhejiang University Medical Ethics Committee (2019–058). Participation in the study was voluntary. The head nurse of the NICU checked the information of preterm infants and their mothers upon hospital admission to screen the mothers’ eligibility and then provided details about the study to the mothers. The researcher provided a full explanation to the potential participants after obtaining mothers’ consent through their provision of their contact information to the researcher. All information provided by the participants to the researcher were stored on an encrypted hard disk, which was placed in a safe, separate location. All names in the study were pseudonyms.

## Results

A total of 23 participants (labeled A–W), aged 24–42, participated in the study, and the gestational age of preterm infants ranged from 29^+ 3^ weeks to 35^+ 5^ weeks. The demographic characteristics of participants and preterm infants were shown in Table 1.

**Table 1:** Demographic Characteristics of Participants (*N* = 23)

Characteristic	n (%)
**Maternal age**	
≤ 30 years	10 (43.5%)
31–35 years	7 (30.4%)
36–40 years	4 (17.4%)
>40 years	2 (8.7%)
**Delivery mode**	
Caesarean	17 (73.9%)
Vaginal	6 (26.1%)
**Parity**	
First	13 (56.5%)
Second	7 (30.4%)
≥ Third	3 (13.0%)
**Gestational age**^a^	
≤ 30 weeks	2 (8.7%)
30^+ 1^ - 32 weeks	6 (26.1%)
32^+ 1^ - 34 weeks	12 (52.2%)
> 34 weeks	3 (13.0%)
**Birth weight of infants (*N*** **= 25)**^a^	
≤ 1500 g	6 (24.0%)
1501 – 2000 g	16 (64.0%)
> 2000 g	3 (12.0%)
**Singleton/ Multiparity**	
Singleton	21 (91.3%)
Multiparity	2 (8.7%)
**Educational level**^b^	
High school	1 (4.3%)
Postsecondary qualification	6 (26.1%)
Bachelor’s degree or above	16 (69.6%)
**Place of residence**	
Village/town	5 (21.7%)
City	18 (78.3%)

^a^N = 25 due to two cases of twin births

^b^None of the participants had a Grade 9 or lower education

Three main themes (Table 2) regarding out-of-hospital human milk management were identified: 1) awareness of human milk management and a willingness to adopt it; 2) lack of standardization regarding expressing, storing, and transporting expressed human milk; and 3) the need for more external support.

**Table 2:** Data Analysis Structure

Theme	Theme Definition	Category	Category Definition
Awareness of human milk management and a willingness to adopt it	This theme describes the participants’ perceptions and attitudes toward human milk management out of the hospital and that these perceptions and attitudes can be changed.	1 Perceptions and attitudes of human milk management 2 Mothers’ original perceptions and attitudes changed.	1 Descriptions of objective information related to human milk management 2 Descriptions of the subjective evaluation of human milk management and the resulting behavioral tendencies
The lack of standardization regarding expressing, storing, and transporting expressed human milk	This theme relates to the participants’ experiences in expressing, storing, and transporting expressed human milk.	1 Preparations before human milk expression 2 Devices for expressing human milk 3 Knowledge and understanding	1 Descriptions of cleaning and preparation before human milk expression 2 Descriptions of milk expression equipment and milk storage devices 3 Descriptions of any knowledge related to the human milk management process
The need for more external support	This theme describes the sources of support received and more external support is needed.	1 Descriptions of the sources of external support received. 2 Descriptions of the need of receiving more external support	1 Descriptions of sources that provided helpful information or action 2 Descriptions of desired information and action

### Theme 1: awareness of human milk management and a willingness to adopt it

All participants said that they recognized that human milk is the most ideal food for infants. They indicated their willingness to “do the best” to provide human milk for their infants. One participant mentioned:

*“I know human milk is good for babies. My best friend has told me that since I got pregnant. Now I can’t be with my baby, but I hope my milk will connect me to my baby.”* ***(T).***

For a small number of mothers, the awareness of providing human milk was established after their interactions with medical staff or peers. Two participants described the increased awareness of human milk expression as follows:

*“I had never wanted to breastfeed. This is not because the baby is hospitalized. I heard from the doctors and nurses that the colostrum is particularly good. The human milk for the seven days before the birth of the baby is colostrum. I want to feed the colostrum to my baby. I hope the baby will recover faster.”* ***(H).***

*“At first, I didn’t want to provide human milk. It was too troublesome, but the mothers in the WeChat group said that human milk is good. If we have any questions we don’t understand, just ask in the group and see what others do. Then I started collecting and providing human milk.”* ***(D).***

### Theme 2: lack of standardization regarding expressing, storing, and transporting expressed human milk

Many participants experienced a lack of standardization in the implementation of expressing, storing, and transporting expressed human milk.

#### Sub-theme I: differentiation of preparations before human milk expression

All participants knew that they needed to conduct self-cleaning and breast pump-cleaning before expressing human milk after health education, but some details that not mentioned in health education manual were still different. Some participants mentioned how they conduct self-cleaning.

*“I wash my hands every time before expression, sometimes with tap water, sometimes with soap.”* ***(F)***.

*“Sometimes my breasts are wiped with water, sometimes not.”* ***(D).***

*“I do some cleaning work first, wash my hands and wipe the nipples with the ethanol cotton ball bought from a pharmacy, and then wipe it with clean water.”* ***(M).***

Some participants mentioned how they conduct equipment-cleaning.

*“I sterilize the breast pump with a sterilizer.”* ***(P)***.

*“I sterilize the breast pump in boiling water.”* ***(E).***

#### Sub-theme II: differentiation of devices for expressing human milk

The NICU ward and human milk bank only did not provide mothers with human milk expression devices. All participants had to purchase milk expression equipment, complete human milk expression at home, and then transport the expressed human milk to the hospital for use by the infant. The statement on breast pump selection in the health education manual reads “We recommend using an electric, hospital-grade breast pump”. Participants often chose breast pumps and milk storage devices based on their personal needs.

*“I bought a single-sided breast pump, and I don’t need to use my hands to express milk. The electric frequency can also be adjusted. It is more convenient and flexible to use than manually.”* ***(L).***

*“I chose a bilateral breast pump. Milking both breasts at the same time.”* ***(S).***

#### Sub-theme III: insufficient knowledge and understanding

The medical staff explained the infant’s condition and provided health education to the father in most cases because mother’s inconvenience after childbirth, and then the father relayed information on milk management to the mother. One participant ***(T)*** said, *“I don’t know what the nurse said specifically at the time. My husband told me that the nurse asked us to follow the hospital’s public account on WeChat and read the (milk management) manual on it.”* Even with health education about expressing knowledge in the manual, several participants did not have ehough knowledge about human milk expression—for example, not knowing when milk collection starts:

*“I don’t know when to start the expression or collection. I didn’t have human milk and couldn’t express milk for the first two or three days. I waited until the fourth day when I felt a little swelling in my breasts before I started expression.”* ***(T).***

Their knowledge about the frequency of milk expression was insufficient, and one participant did not express milk at night and another participant suspended expressing in the first few days.

*“I usually collect six times a day, about 15 minutes at a time. I don’t collect human milk at night because I don’t feel bloated.”* ***(L).***

*“My breasts didn’t feel ‘full’ and I could express a little milk in the first few days, but then I lost milk. I stopped expressing milk.”* ***(A).***

Participants were not sure whether their own management measures could guarantee the quality of their milk.

*“I put the human milk in a plastic bag with some ice cubes in it. My home is next to the hospital. Just send it over, there should be no problem.”* ***(T)***.

*“Based on the hospital’s request, I bought the milk storage bag, but after the milk storage bag was filled with human milk, the air was not expelled. No one told me what to do, and I don’t know if this is feasible.”* ***(H).***

### Theme 3: the need for more external support

Participants expected to receive professional guidance and help from medical staff to maintain human milk management awareness and enhance human milk management practices.

*“I don’t know what kind of human milk storage bag is suitable, where to buy it, how long can human milk be stored, and I don’t know if the baby will have other problems with this kind of human milk. I just do it depending on what others do. I would certainly appreciate professional guidance (to allay my concerns).”* ***(Q).***

Participants, after their delivery, received a health education manual on human milk management that was provided by the nurses. They also sought for help from the Internet, books, and communication among themselves. Some participants have shown researchers their access to information. Most books were professional and scientifically based, however the quality of some information from peers and web could not be approved.

*“After I had a baby, the nurse told me what to do. I also bought a few baby-care books to read. Occasionally I encounter problems I don’t know, I just Baidu (a search engine commonly used in China) it.”* ***(E).***

Due to the traditional custom of *sitting month*, participants needed support from family members with their human milk management. The distance between home and hospital could also affect milk management, for example, a mother’s home is only about 3 km away from the hospital and this mother transported human milk daily. Another mother even needed to transport human milk across cities, which took at least 50–70 minutes one time. Her family transported frozen human milk every 3 days.

*“I want to transport human milk, but my home is really far away from hospital. It took several bus rides back and forth to get to the hospital, and my family members are very busy. I am ‘sitting the month’ at home and can only deliver it occasionally, and I hope someone in the family can help me transport human milk regularly.”* ***(V).***

## Discussion

This study explored how mothers manage human milk out of the hospital, which revealed their ideas on management through three themes. From the results of this study, in facilities we saw women understanding the importance of the milk for their babies and trying to express it with peer and professional support. On the other hand, difficulties in lack of standardization regarding human milk expressing, storing and transportation seem to have bothered mothers, and more support is needed after mothers’ discharge. Similar findings have been reported in other studies on expressing human milk to feed infants [27, 28].

A human milk management manual was provided by NICU and the participants indicated that they received this manual when their infants were admitted to NICU. Researchers of this study found that just having manual, even if the manual came from the hospital’s official sources, did not guarantee that the recommendations were well communicated and implemented, indicating that conducting out-of-hospital human milk management education on admission and written education alone were insufficient.

This study found that mothers differed in the details of how they expressed, stored, and transported expressed human milk. This finding is consistent with an earlier study [29]. According to the *Protecting, Promoting and Supporting Breastfeeding in Facilities Providing Maternity and Newborn Services* guidelines, “There was no evidence that a particular type of pump was associated with a higher level of milk contamination, infant sepsis or transfer to feeding at the breast” [30]. The difference in pumps may not affect the quality of expressed human milk. However, how the pump is sterilized is a factor that affects the quality of expressed human milk [31]. Studies have reported that at least 52% of mothers had one or more experiences of ineffective sterilization of breast pump collection kits [32] and only 54% of mothers correctly followed the information on the refrigeration times of expressed human milk [17]. This may increase the risk of bacterial contamination of human milk expressed through a breast pump.

The main information for 22% of mothers comes from unofficial sources—for example, websites—and these resources may not recommend the same methods as the official guidelines [17]. The Internet and peer instruction were also other important sources of knowledge for participants in this study, but the scientific nature of the information from these channels was not certain. This caused concerns, especially for those participants who wished to provide fresh human milk to their preterm infants. We further found that participants hope to receive more guidance from health professionals and help from their families to maintain the awareness of human milk management and ensure its smooth implementation. Mothers are generally inclined to rate their spouse or partner as supportive in the breastfeeding process, with smaller proportions indicating supportiveness of their closest friend or their own mother [33].

This expectation of lactation support should be better met to ensure that every mother receives accurate, comprehensive, evidence-based lactation support while separated from their infant. Not only is it necessary to provide a detailed and standardized routine for human milk management, but more importantly, the routine must be accepted and understood by mothers. Using evidence-based science about human milk and disseminating this information to mothers and their families should become an established institutional culture. Continuous health education and supporting measures should be promoted. For example, both parents should be ideally informed about human milk’s information including details at the prenatal consultation with the medical staff, and a health education kit with human milk management instructions and practical information is handed out at the same time [34]. It is also important to ensure that human milk management processes are consistent with current best practices, while providing continuous professional support. The US Centers for Disease Control and Prevention (CDC) now recommends that mothers should use an additional method of breast pump decontamination, especially for feeding vulnerable infants, such as those born preterm. This update followed a case of bacterial infection in a preterm infant due to a contaminated breast pump [35]. Furthermore, NICU and Maternity Unit could provide lactation support such as ready-to-use electric breast pumps [36]. So that the details could be noticed and shared when mothers’ expressing milk with medical staff’s presence. In addition to medical staff’s specific knowledge in helping family possessing skills on human milk management and breastfeeding preterm infants [37], positive attitude towards human milk and provide information and practical guidance to family in a sensitive, flexible and timely manner also are major facilitators of milk management [38]. Although daily provision of human milk is expected. For families who cannot transport human milk daily for reasons, such as distance, the NICU recommended them to transport frozen human milk every 3 days.

The presence of lactation support providers in the NICU increased the number of direct breastfeeding sessions on the day of discharge. Moreover, the breastfeeding rate after discharge from the hospital has been positively correlated with the participation of International Board-Certified Lactation Consultant (IBCLC) support [39]. More well-trained staff—for example, NICU nurses and IBCLCs—could continue to provide mothers and families with conflict-free (suggestions and appropriate advice) and may improve the situation, which has a positive effect on maintaining breastfeeding. The type of guidance could be diversified, and services could be provided until the mother fully understands the information or no longer needs it.

### Study limitations

Although a maximum variation sampling strategy was used, this study was conducted in only one NICU in China, in which parents could not care for their infants. The findings might not be applicable to mothers whose infants were hospitalized in other settings. In addition, most of the participants were well-educated. It cannot be assumed that the findings apply to mothers with less education.

## Conclusion

In conclusion, this study showed that many mothers had questions about the implementation of out-of-hospital human milk management and would welcome professional advice and guidance. These findings suggest the necessity of providing continuous out-of-hospital human milk management guidance and providing direction for revising and developing current health education.

## Supplementary Information

**Additional file 1.**

## Data Availability

The datasets and transcripts of the current study can be obtained from the corresponding author on reasonable request.

## Abbreviations

IBCLC: International Board-Certified Lactation Consultant
NICU: Neonatal Intensive Care
UNICEF: United Nations Children’s Fund
WHO: World Health Organisation.
