# Supplementary material for: Experiences of mothers of NICU preterm infants in milk management out of the hospital: a qualitative study
Source: Int Breastfeed J. 2022 Dec 31;17:95. doi: 10.1186/s13006-022-00540-2 (PMC9805215; doi:10.1186/s13006-022-00540-2)
Supplement: Supplementary file 1 — Additional file 1. [file 13006_2022_540_MOESM1_ESM.docx]

**Supplemental Material**

**Interview Guide**

1. Have you learned about breast milk expression, storage, and transfer?
2. How do you feel about that?
3. How did you receive this information?
4. How did your express, store, and transfer breast milk? Have you had any difficulties?
5. Tell me more about how you handled it.
6. When did you start to worry about the expression, storage, and transfer of breast milk?
7. Did you try anything to help you improve the expression, storage, and transfer of breast milk?
8. Do you think you can master the management of breast milk by yourself?
   1. If not, what support did you receive?
   2. What support did your family give you?
   3. Do you think the hospital can provide better support and assistance for the expression, storage, and transfer of breast milk?
